# Improving large language models for clinical named entity recognition via prompt engineering

**DOI:** 10.1093/jamia/ocad259

**Published:** 2024-01-27

**Authors:** Yan Hu, Qingyu Chen, Jingcheng Du, Xueqing Peng, Vipina Kuttichi Keloth, Xu Zuo, Yujia Zhou, Zehan Li, Xiaoqian Jiang, Zhiyong Lu, Kirk Roberts, Hua Xu

**Affiliations:** McWilliams School of Biomedical Informatics, Houston, TX, United States; Section of Biomedical Informatics and Data Science, School of Medicine, Yale University, New Haven, CT, United States; National Center for Biotechnology Information, National Library of Medicine, National Institutes of Health, Bethesda, MD, United States; McWilliams School of Biomedical Informatics, Houston, TX, United States; Section of Biomedical Informatics and Data Science, School of Medicine, Yale University, New Haven, CT, United States; Section of Biomedical Informatics and Data Science, School of Medicine, Yale University, New Haven, CT, United States; McWilliams School of Biomedical Informatics, Houston, TX, United States; McWilliams School of Biomedical Informatics, Houston, TX, United States; McWilliams School of Biomedical Informatics, Houston, TX, United States; McWilliams School of Biomedical Informatics, Houston, TX, United States; National Center for Biotechnology Information, National Library of Medicine, National Institutes of Health, Bethesda, MD, United States; McWilliams School of Biomedical Informatics, Houston, TX, United States; Section of Biomedical Informatics and Data Science, School of Medicine, Yale University, New Haven, CT, United States

**Keywords:** prompt engineering, large language models, clinical named entity recognition, GPT-3.5, GPT-4

## Abstract

**Importance:**

The study highlights the potential of large language models, specifically GPT-3.5 and GPT-4, in processing complex clinical data and extracting meaningful information with minimal training data. By developing and refining prompt-based strategies, we can significantly enhance the models’ performance, making them viable tools for clinical NER tasks and possibly reducing the reliance on extensive annotated datasets.

**Objectives:**

This study quantifies the capabilities of GPT-3.5 and GPT-4 for clinical named entity recognition (NER) tasks and proposes task-specific prompts to improve their performance.

**Materials and Methods:**

We evaluated these models on 2 clinical NER tasks: (1) to extract medical problems, treatments, and tests from clinical notes in the MTSamples corpus, following the 2010 i2b2 concept extraction shared task, and (2) to identify nervous system disorder-related adverse events from safety reports in the vaccine adverse event reporting system (VAERS). To improve the GPT models' performance, we developed a clinical task-specific prompt framework that includes (1) baseline prompts with task description and format specification, (2) annotation guideline-based prompts, (3) error analysis-based instructions, and (4) annotated samples for few-shot learning. We assessed each prompt's effectiveness and compared the models to BioClinicalBERT.

**Results:**

Using baseline prompts, GPT-3.5 and GPT-4 achieved relaxed F1 scores of 0.634, 0.804 for MTSamples and 0.301, 0.593 for VAERS. Additional prompt components consistently improved model performance. When all 4 components were used, GPT-3.5 and GPT-4 achieved relaxed F1 socres of 0.794, 0.861 for MTSamples and 0.676, 0.736 for VAERS, demonstrating the effectiveness of our prompt framework. Although these results trail BioClinicalBERT (F1 of 0.901 for the MTSamples dataset and 0.802 for the VAERS), it is very promising considering few training samples are needed.

**Discussion:**

The study’s findings suggest a promising direction in leveraging LLMs for clinical NER tasks. However, while the performance of GPT models improved with task-specific prompts, there's a need for further development and refinement. LLMs like GPT-4 show potential in achieving close performance to state-of-the-art models like BioClinicalBERT, but they still require careful prompt engineering and understanding of task-specific knowledge. The study also underscores the importance of evaluation schemas that accurately reflect the capabilities and performance of LLMs in clinical settings.

**Conclusion:**

While direct application of GPT models to clinical NER tasks falls short of optimal performance, our task-specific prompt framework, incorporating medical knowledge and training samples, significantly enhances GPT models' feasibility for potential clinical applications.

## Introduction

Electronic health records (EHRs) contain a vast quantity of unstructured data, including clinical notes, which can offer valuable insights into patient care and clinical research.^1^ However, manually extracting pertinent information from clinical notes presents a challenge, as it is labor-intensive and time-consuming. To address these challenges, researchers have developed various natural language processing (NLP) techniques for automating the clinical information extraction process. Clinical named entity recognition (NER) is a critical clinical NLP task focusing on recognizing boundaries of clinical entities (ie, words/phrases) and determining their semantic categories, such as medical problems, treatment, and tests.^2^ With the help of advancements in clinical NER, the time and effort required for manual chart review and coding by health professionals can be significantly reduced, thus improving patient care efficiency, and accelerating clinical research.^3^

Early clinical NER systems often depend on predefined lexical resources and syntactic/semantic rules derived from extensive manual analysis of text.^4^ Over the past decade, machine learning-based approaches have gained popularity in clinical NER research.^5^ Current popular clinical information extraction systems, such as cTAKES and CLAMP, are hybrid systems that integrate rule-based and machine learning-based techniques.^6^ Nevertheless, a bottleneck in building machine learning-based clinical NER models is to develop large, annotated corpora, which often require domain experts and take a long time to build. More recently, transformer-based large language models (LLMs) have emerged as the leading method for developing clinical NLP applications. Bidirectional Encoder Representations from Transformers (BERT) is a widely used pretrained language model that learns contextual representations of free text.^7^ Utilizing BERT as the foundation, domain-specific language models like BioBERT, PubMedBERT (trained on biomedical literature), and ClinicalBERT (trained on the MIMIC-III dataset) have been further developed.^8–10^ These models have been applied to clinical NER tasks via transfer learning (ie, fine-tuning the models on clinical NER corpora), and have shown improved performance with fewer annotated samples.^8–10^

Generative Pre-trained Transformers (GPT) represent another type of LLM capable of generating human-like responses based on textual input. In November 2022, OpenAI unveiled GPT-3.5,^11^ a groundbreaking language model that quickly garnered interest from researchers and technology enthusiasts. As an extension of GPT-3, GPT-3.5 serves as a conversational agent adept at following complex instructions and generating high-quality responses across various scenarios. Besides its conversational skills, GPT-3.5 has exhibited remarkable performance in many other NLP tasks, such as machine translation and question-answering,^12^ even in zero-shot or few-shot learning scenarios,^13^ where the model can be applied to new tasks without using annotated samples or with a very small number of annotated samples. On March 18, 2023, OpenAI released GPT-4, one of the most advanced NLP models at the time, which has demonstrated even greater capabilities and performance improvements over GPT-3.5.^14^

As interest in GPT models continues to surge, numerous studies are currently exploring the wide range of possibilities offered by these LLMs. One prominent example of GPT models for medicine is that GPT-3.5 passed the US medical license exam with about 60% accuracy, which has further sparked the potential use of GPT-3.5 and GPT-4 in the medical domain.^15^ More applications of GPT-3.5 and GPT-4 in healthcare have also been discussed.^16–23^ With those motivations, this study aims to investigate the potential of GPT models for clinical NER tasks.

Meanwhile, prompt engineering has emerged as a crucial aspect of utilizing GPT models effectively for various NLP tasks. Prompt engineering involves designing input prompts that guide the model to generate desired outputs, thereby improving its performance on specific tasks.^24^ Several studies have explored prompt engineering for GPT models in open-domain settings, demonstrating its effectiveness in enhancing the model's performance across a range of tasks.^25^^,^^26^ In the biomedical domain, some work has been done on prompt engineering for GPT models, focusing on tasks such as biomedical question-answering, text classification, and NER.^27–29^ However, to the best of our knowledge, no work has been conducted on prompt engineering for GPT models specifically targeting NER tasks in clinical texts. This highlights the need for further investigation into the potential of GPT models and prompt engineering techniques for clinical NER applications.

The contributions of this study are 3-fold. First, we proposed a prompt framework for clinical NER by incorporating entity definitions, annotation guidelines, and annotated samples, and demonstrated its effectiveness on 2 NER tasks (eg, improving the performance of the GPT models by up to ∼20% and making it more competitive to fine-tuned models such as BioClinicalBERT). Second, we discussed how the recent LLMs such as GPT models will change the development of NER systems in the medical domain. This is important because LLMs show a great potential for developing generalizable clinical NER systems without substantial annotation efforts. Finally, this study also established a novel benchmark to evaluate the performance of the LLMs, GPT-3.5 and GPT-4, for the task of clinical NER. We leveraged 2 distinct clinical NER tasks as benchmarks, namely the 2010 i2b2 concept extraction task^30^ and the nervous system disorder-related event extraction task.^31^ All code and datasets are made publicly available to the community.

## Methods

### Task overview

This study mainly aims to quantify the capabilities of GPT-3.5 and GPT-4 for the clinical NER tasks, as defined in the 2010 i2b2 concept extraction task^30^ and the nervous system disorder-related event extraction task,^31^ and propose clinical task-specific prompts to improve their performance. The i2b2 concept extraction task involves identifying and classifying key medical concepts such as problems, treatments, and tests from patient reports, for example, recognizing “lung cancer” as a problem, “chemotherapy” as a treatment, and “CT scan” as a test. The nervous system disorder-related events extraction task, on the other hand, aims to extract nervous system disorder-related events from safety reports in the vaccine adverse event reporting system (VAERS). Unlike typical EHRs which provide comprehensive patient histories and treatment details, the VAERS dataset is primarily focused on postvaccination adverse events, for example, recognizing “neurological exam” as an investigation and “tremors” as a nervous adverse event. The MTSamples dataset was used for the first task, and the VAERS dataset was used for the second task (see details in the “Datasets” section).

**Figure 1.**
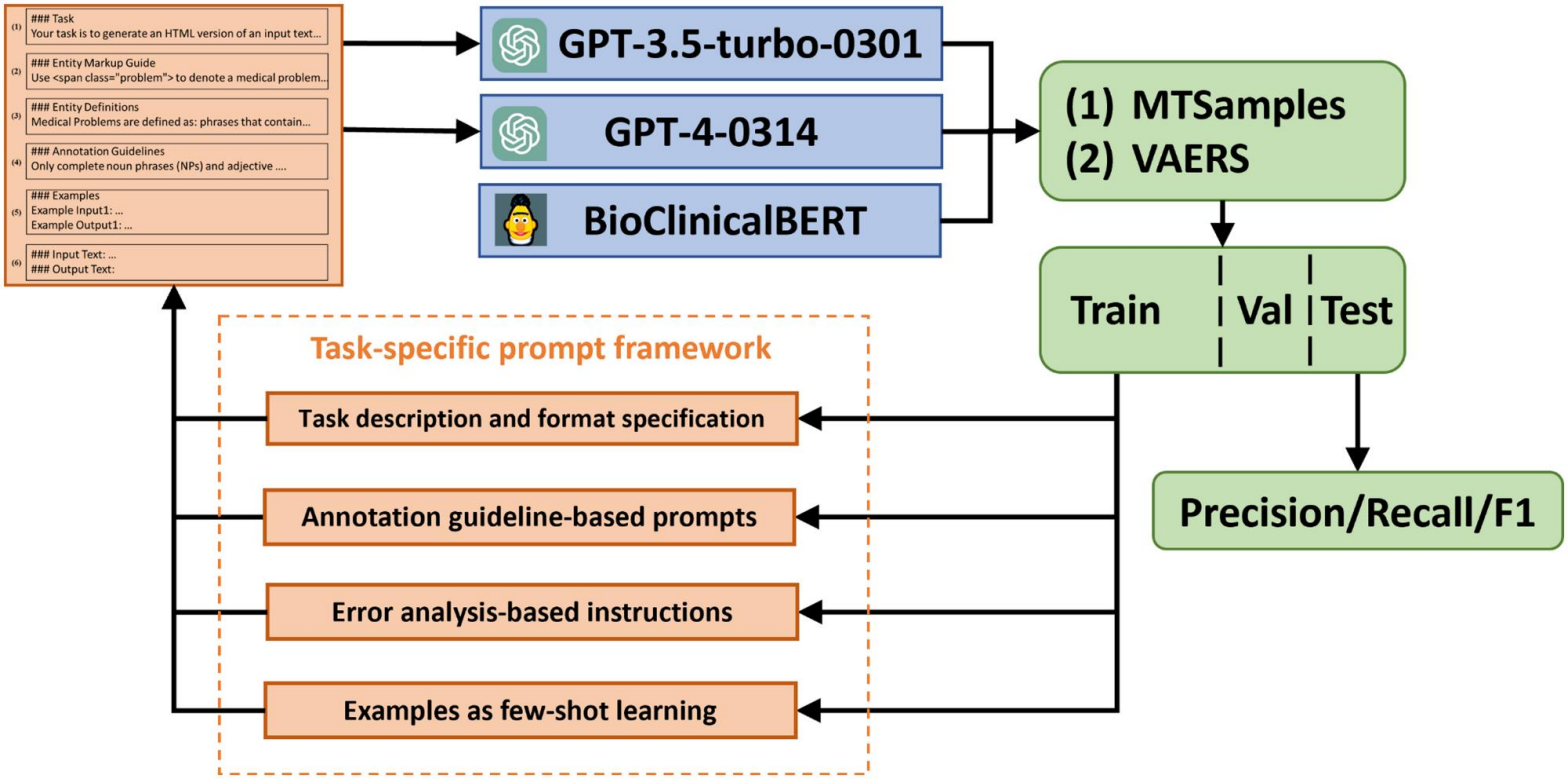
An overview of the study workflow.

The prompt components used for GPT models and the primary workflow of our study are depicted in Figure 1. We proposed task-specific prompts (see details in the “Prompt engineering” and “Evaluation” sections) to both GPT-3.5 and GPT-4 on the 2 tasks and evaluated the performance based on the output. Error analysis was then performed on the training set to identify prevalent errors, and error analysis-based instructions targeting these errors were added to help the models correct the errors. Performance of different models was finally evaluated using the independent test set.

### Datasets

Two clinical NER datasets were used in our study, including (1) MTSamples, a set of 163 fully synthetic discharge summaries from MTSamples, which was annotated according to the annotation guidelines from the 2010 i2b2 challenge, which aims at extracting Medical Problem, Treatment, and Test,^30^ and (2) the VAERS corpus, a set of 91 publicly available safety reports in VAERS, aiming at extracting nervous system disorder-related events.^31^

The MTSamples dataset is fully synthetic, meaning that it has been artificially generated and contains no real patient information. The VAERS dataset, on the other hand, is derived from publicly available postmarket safety reports that are anonymized and do not contain personally identifiable information. So, no sensitive data were sent to OpenAI API, making this study free of privacy concerns. Upon consultation, it was determined that our study did not require IRB approval.

The 2 datasets were split into training, validation, and test subsets. The training and validation subsets served the purpose of fine-tuning the BioClinicalBERT model. Annotated samples in prompts were randomly sampled from the training sets. The training sets were also used for error analysis to optimize our prompt strategies. The test subsets, however, were reserved exclusively for evaluating the final performance and for comparative analysis. A descriptive statistic of entities in these datasets is presented in Table 1.

**Table 1.** Dataset statistics utilized in this study.

Datasets	Entities	Train	Valid	Test	Total
MTSamples	Medical problem	538	203	199	940
	Treatment	149	43	35	227
	Test	120	39	50	209
VAERS	Investigation	148	29	59	236
	Nervous adverse event	406	83	162	651
	Other adverse event	301	62	167	530
	Procedure	338	57	126	521

Abbreviation: VAERS, vaccine adverse event reporting system.

### Models

We fine-tuned NER models using BioClinicalBERT,^32^ to serve as baselines of traditional supervised learning approaches. We present results for supervised learning on both the MTSamples test set and the VAERS test set. The model weights were initialized using the transformers package, available at https://huggingface.co/emilyalsentzer/Bio_ClinicalBERT.^32^^,^^33^ The hyperparameters employed during model training included a learning rate of 5e−5, a training batch size of 4, 20 epochs, and a weight decay of 0.01 using the AdamW optimizer.^34^ In addition to fine-tuning NER models using BioClinicalBERT, we also employed a traditional machine learning approach for comparison. We utilized a Conditional Random Field (CRF) model with word features, including Bag-of-word, capitalization of letters in words, and prefixes and suffixes of words.^35^

Regarding the GPT models, we used the specific versions GPT-3.5-turbo-0301 and GPT-4-0314 for reproducibility. Temperature in a generative language model refers to a parameter that controls the randomness in the model's predictions, typically ranging from 0 (completely deterministic) to 1 or higher (increasingly random and diverse outputs). The temperature parameter for GPT models was set to 0 to minimize randomness in response generation. A lower temperature value restricts the model's tendency to take creative leaps, thereby ensuring more predictable and consistent outputs. This is crucial in clinical NER tasks where accuracy and reliability of information extraction are paramount. In our setup, the GPT models were interacted with in a “user” role. This role simulates a real-world user interaction with the model, where the “user” inputs prompts and the model generates responses accordingly. This approach reflects a typical use-case scenario for these models in practical applications. All input and output datasets along with prompt variants are included with Jypter notebooks that can interface with the OpenAI API in our GitHub repository. At the time of this study, costs of GPT-3.5 per 1k tokens were approximately $0.03 for input and $0.06 for output. Costs of GPT-4 per 1k tokens were approximately $0.001 for input and $0.002 for output. Because of privacy issues, notes containing Personal Identifiable Information (PII) could not be used in this experiment and should not be used with the GPT API.

### Prompt engineering

For GPT models, we proposed a task-specific prompt including the following components:

1. Baseline prompt with task description and format specification: This component provides the LLMs with basic information about the tasks we are instructing them to perform and in what format the LLMs should output results. We instructed the models to highlight the named entities within an HTML file using <span> tags with a class attribute indicating the entity types. This allows the output from GPT models to be easily converted into a traditional Inside-Outside-Beginning format, which allows for a direct comparison of NER performance with findings from existing studies.
2. Annotation guideline-based prompts: This component contains entity definitions and linguistic rules derived from annotation guidelines. Entity definitions offer comprehensive, unambiguous descriptions of an entity within the context of a given task. They play an instrumental role in steering the LLM toward the precise identification of entities within text documents. We noticed that the model's predictions often differed substantially from the gold standard in terms of grammatical structure. For example, discrepancies may arise concerning what types of phrases to be included (eg, noun phrases or adjective phrases). To enhance the model's performance, we referred to and incorporated rules in the annotation guidelines to address these issues.
3. Error analysis-based instructions: In addition to the original annotation guidelines, we also incorporated additional guidelines following error analysis of GPT outputs using the training data. For example, we noticed that GPT models often tend to annotate consultation procedures as test entities. To prevent this, we incorporated a specific rule stating, “Consultation procedures should not be annotated as tests.”
4. Annotated samples: To further assist the LLMs in understanding the task and generating accurate results, we provided a set of annotated samples to improve its performance in a few-shot learning setting. We randomly selected either 1 or 5 annotated examples (1- or 5-shot learning) from the training set and formatted them according to the task description and entity markup guide.
  For instance, given a sentence “He had been diagnosed with osteoarthritis of the knees and had undergone arthroscopy years prior to admission,” with “osteoarthritis of the knees” and “arthroscopy” annotated as medical problem and test entities, we incorporated this sentence into the prompt using the following format:
  ### Examples
  Example Input: He had been diagnosed with osteoarthritis of the knees and had undergone arthroscopy years prior to admission.
  Example Output: He had been diagnosed with <span class=“problem”>osteoarthritis of the knees</span> and had undergone <span class=“test”>arthroscopy</span> years prior to admission.

We compared the effectiveness of different prompt components by incrementally incorporating annotation guideline-based prompts, error analysis-based instructions, and annotated samples as shown in Table 2 (see the complete prompts for 2 datasets in Supplementary Material S1.1).

**Table 2.** An Illustration of the prompt framework for clinical NER.

Prompt types	Examples
(1) Baseline prompts	### Task Your task is to generate an HTML version of an input text, marking up specific entities related to healthcare. The entities to be identified are: “medical problems,” “treatments,” and “tests.” Use HTML <span> tags to highlight these entities. Each <span> should have a class attribute indicating the type of the entity. ### Entity Markup Guide Use <span class=“problem”> to denote a medical problem…
(2) Annotation guideline-based prompts	### Entity Definitions Medical Problems are defined as: phrases that contain observations made by patients or clinicians about the patient’s body or mind that are thought to be abnormal or caused by a disease… ### Annotation Guidelines: Only complete noun phrases (NPs) and adjective phrases (APs) should be marked. Terms that fit concept semantic rules, but that are only used as modifiers in a noun phrase should not be marked…
(3) Error analysis-based instructions	### Error-analysis-based Guidelines: Consultation procedures should not be annotated as tests…
(4) Annotated samples via few-shot learning	### Examples Example Input1: He had been diagnosed with osteoarthritis of the knees and had undergone arthroscopy years prior to admission. Example Output1: He had been diagnosed with <span class=“problem”>osteoarthritis of the knees</span> and had undergone <span class=“test”>arthroscopy</span> years prior to admission…

Abbreviation: NER, named entity recognition.

### Evaluation

The performance of the models was evaluated using Precision (P), Recall (R), and F1 scores, following the same evaluation script in the 2010 i2b2 challenge.^30^ These scores were computed based on both exact-match and relaxed-match criteria. In the context of an exact match, an extracted entity should have identical token boundary and entity type as that in the gold standard. For relaxed match, an extracted entity that exhibits overlap in text and shares the same entity type with the gold standard is acceptable.

## Results

### Zero-shot performance with different prompts

**Figure 2.**
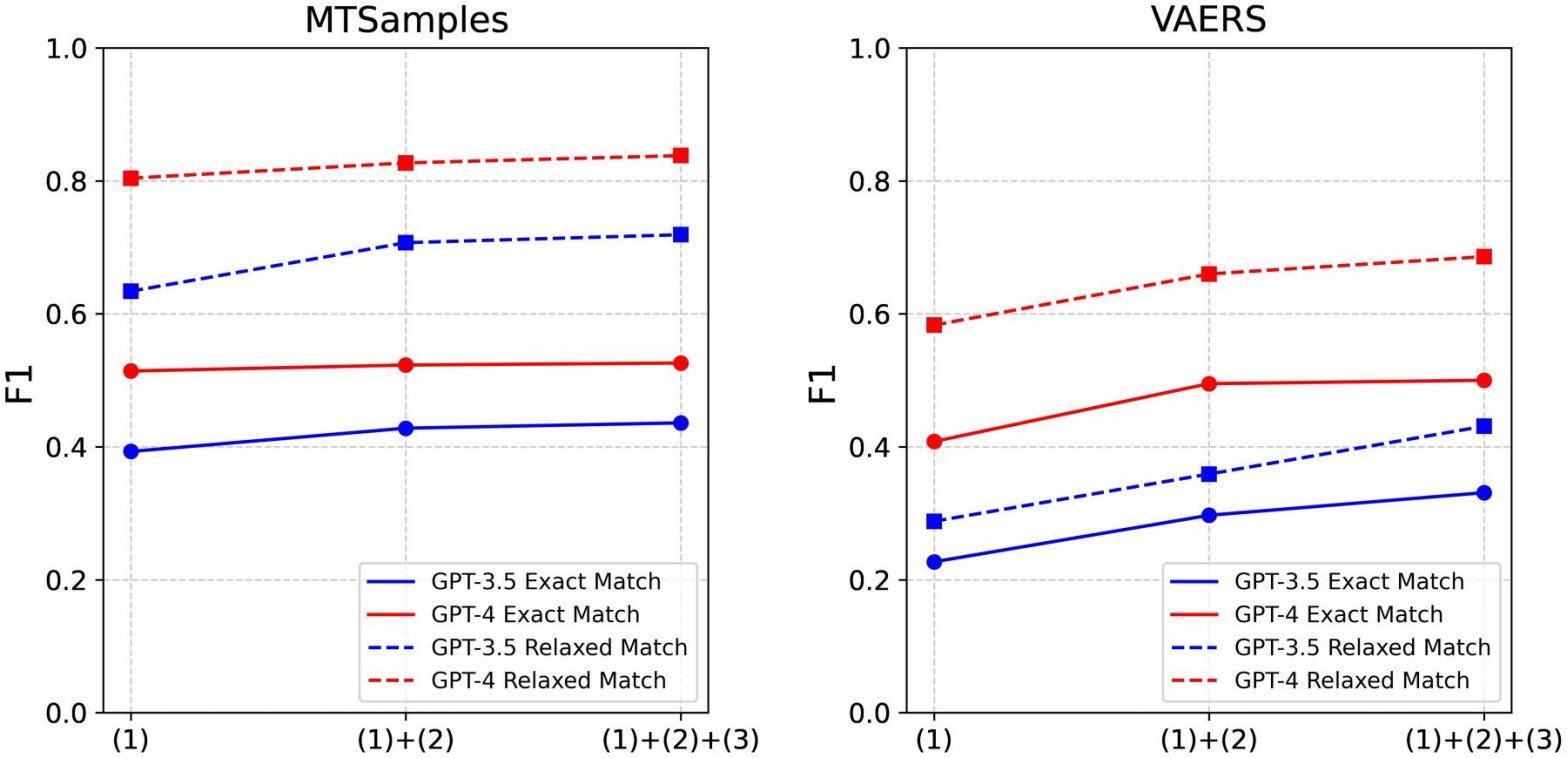
Performance comparison using different prompt strategies.

The performance evaluation of GPT-3.5 and GPT-4 in zero-shot settings using different prompts is detailed in Table 3 and Figure 2. Following the integration of annotation guideline-based prompts and error analysis-based instructions, we noticed an improvement in the performance metrics of both GPT models, across each dataset and under each evaluation criteria. Interestingly, we found these 2 components to have a more pronounced effect on the performance of GPT-3.5 than on GPT-4. More specifically, GPT-3.5 demonstrated an average increase of 0.09 in overall F1 scores, ranging from 0.04 to 0.14. Conversely, GPT-4 displayed a more restrained average improvement of 0.06, with a range of 0.01-0.10. Looking at the dataset-specific effects, these 2 components had a more substantial impact on the VARES dataset compared to the MTSamples dataset. For VARES, we saw an average increase of approximately 0.11, with a range from 0.09 to 0.14. In contrast, for MTSamples, we saw a more modest approximate average increase of 0.04, with the range extending from 0.01 to 0.08.

**Table 3.** Zero-shot performance of GPT-3.5-turbo-0301 and GPT-4-0314 using different prompt strategies.

Models	Prompt strategies	MTSamples						VAERS					
		Exact match			Relaxed match			Exact match			Relaxed match		
		P	R	F1	P	R	F1	P	R	F1	P	R	F1
GPT-3.5	Baseline prompts only (1)	0.492	0.327	0.393	0.794	0.528	0.634	0.510	0.146	0.227	0.626	0.187	0.288
	+ Annotation guideline-based prompts (1)+(2)	0.453	0.405	0.428	0.736	0.680	0.707	0.575	0.200	0.297	0.687	0.243	0.359
	+ Error analysis-based instructions (1)+(2)+(3)	0.462	0.412	0.436	0.755	0.687	0.719	0.569	0.233	0.331	0.730	0.305	0.431
GPT-4	Baseline prompts only (1)	0.486	0.546	0.514	0.762	0.852	0.804	0.420	0.397	0.408	0.599	0.568	0.583
	+ Annotation guideline-based prompts (1)+(2)	0.478	0.577	0.523	0.752	0.919	0.827	0.559	0.444	0.495	0.743	0.593	0.660
	+ Error analysis-based instructions (1)+(2)+(3)	0.488	0.570	0.526	0.777	0.908	0.838	0.536	0.469	0.500	0.727	0.650	0.686

Abbreviation: VAERS, vaccine adverse event reporting system.

### Effect of N-Shot examples on model performance

**Figure 3.**
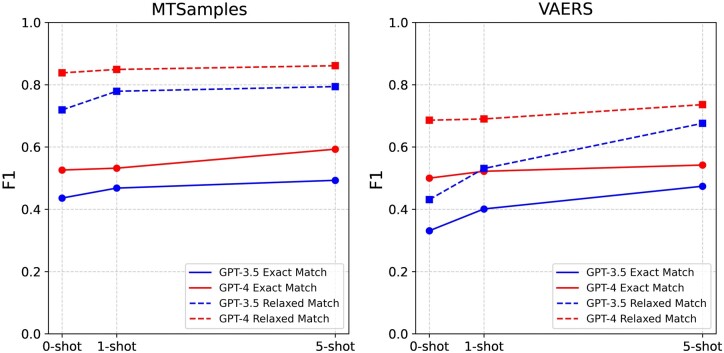
Performance comparison based on different numbers of N-shot examples in each prompt design.

Table 4 and Figure 3 illustrate the performance comparison among different numbers of N-shot examples with all prompt components included. Generally, the inclusion of more examples leads to better model performance. A combination of 5-shot and all prompts produced the best results by GPT-4, achieving F1 0.593 and 0.861 for MTSamples and 0.542 and 0.736 for VAERS under exact and relaxed match, respectively.

**Table 4.** 0-, 1-, and 5-shot performance of GPT-3.5-turbo-0301 and GPT-4-0314 using all prompt components.

Models	Prompt strategies	MTSamples						VAERS					
		Exact match			Relaxed match			Exact match			Relaxed match		
		P	R	F1	P	R	F1	P	R	F1	P	R	F1
GPT-3.5	(1) + (2) + (3)	0.462	0.412	0.436	0.755	0.687	0.719	0.569	0.233	0.331	0.73	0.305	0.431
	+ 1 annotated example (1)+(2)+(3)+(4)	0.475	0.461	0.468	0.779	0.778	0.779	0.561	0.311	0.401	0.733	0.416	0.531
	+ 5 annotated example (1)+(2)+(3)+(4)	0.515	0.472	0.493	0.827	0.764	0.794	0.526	0.432	0.474	0.735	0.626	0.676
GPT-4	(1) + (2) + (3)	0.488	0.570	0.526	0.777	0.908	0.838	0.536	0.469	0.500	0.727	0.650	0.686
	+ 1 annotated example (1)+(2)+(3)+(4)	0.506	0.560	0.532	0.809	0.894	0.849	0.547	0.500	0.522	0.721	0.661	0.690
	+ 5 annotated example (1)+(2)+(3)+(4)	0.555	0.637	0.593	0.804	0.926	0.861	0.513	0.574	0.542	0.701	0.774	0.736

Abbreviation: VAERS, vaccine adverse event reporting system.

### Performance comparison to supervised learning

**Figure 4.**
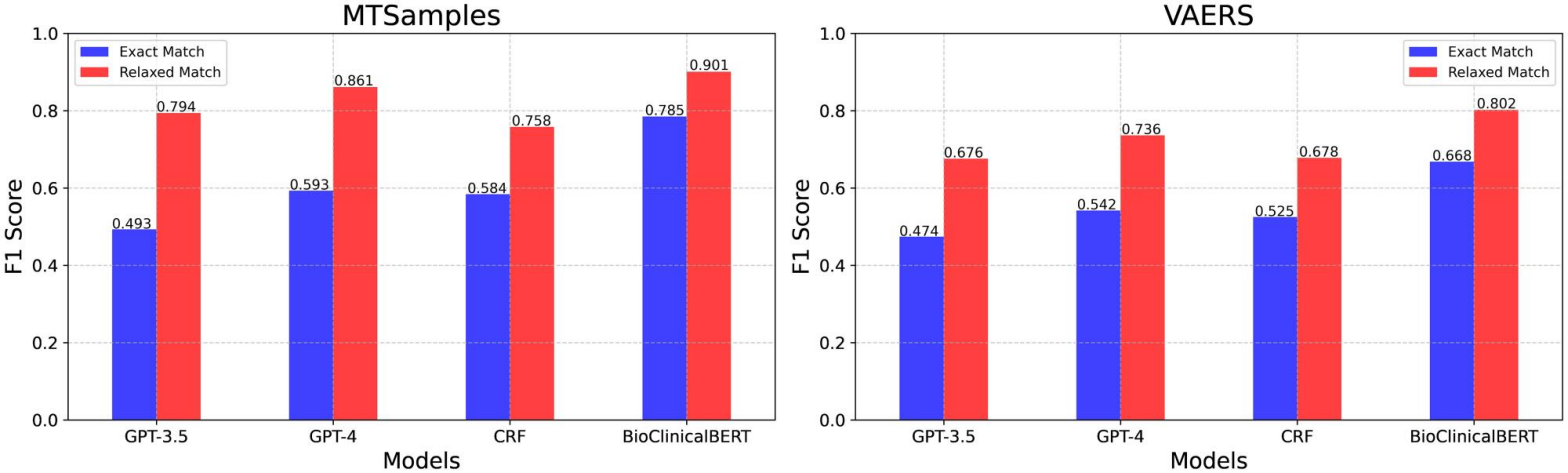
Performance comparison between GPT-3.5, GPT-4, BioClinicalBERT and CRF models.

Table 5 and Figure 4 displays the performance of BioClinicalBERT, CRF, GPT-3.5, and GPT-4 models for comparison. Among the 3 models, BioClinicalBERT still demonstrated the highest performance. For MTSamples, it achieved overall F1 scores of 0.785 and 0.901 under exact match and relaxed match, respectively. Its performance on the VAERS dataset also remained dominant, with overall F1 scores of 0.668 and 0.802 under exact match and relaxed match, respectively. The CRF model achieved an F1 score of 0.584 and 0.525 in MTSamples and VAERS by exact match and surpassing GPT-3.5. In the relaxed-match criteria, the CRF model performed worse than GPT-4 and GPT-3.5 in the MTSamples and had comparable performance to GPT-3.5 in the VAERS dataset. Comparatively, GPT-3.5 lagged on 2 datasets with the lowest performance, yet still demonstrated a decent performance with scores of 0.794 and 0.676, as evaluated by relaxed-match criteria on the 2 datasets respectively. GPT-4 showcased highly competitive performance using the relaxed match criteria, accomplishing F1 scores of 0.861 and 0.736 on the MTSamples and VAERS datasets respectively. It is notable, however, that the performances of GPT-3.5 and GPT-4 as evaluated by the exact-match method were not as impressive as those by the relaxed match. In addition to the test set results, we have provided the model's performance on the validation sets in Supplementary Material S1.2 to ensure the BioClinicalBERT is not overfitting.

**Table 5.** Performance of BioClinicalBERT, CRF, GPT-3.5, and GPT-4 on MTSamples and VAERS datasets.

Model	MTSamples						VAERS					
	Exact match			Relaxed match			Exact match			Relaxed match		
	P	R	F1	P	R	F1	P	R	F1	P	R	F1
GPT-3.5	0.515	0.472	0.493	0.827	0.764	0.794	0.526	0.432	0.474	0.735	0.626	0.676
GPT-4	0.555	0.637	0.593	0.804	0.926	0.861	0.513	0.574	0.542	0.701	0.774	0.736
CRF	0.511	0.681	0.584	0.662	0.887	0.758	0.473	0.591	0.525	0.609	0.764	0.678
BioClinicalBERT	0.785	0.785	0.785	0.915	0.887	0.901	0.698	0.640	0.668	0.846	0.761	0.802

The performance is shown in the order of Precision/Recall/F1.

Abbreviations: CRF, Conditional Random Field; VAERS, vaccine adverse event reporting system.

### Error analysis

**Figure 5.**
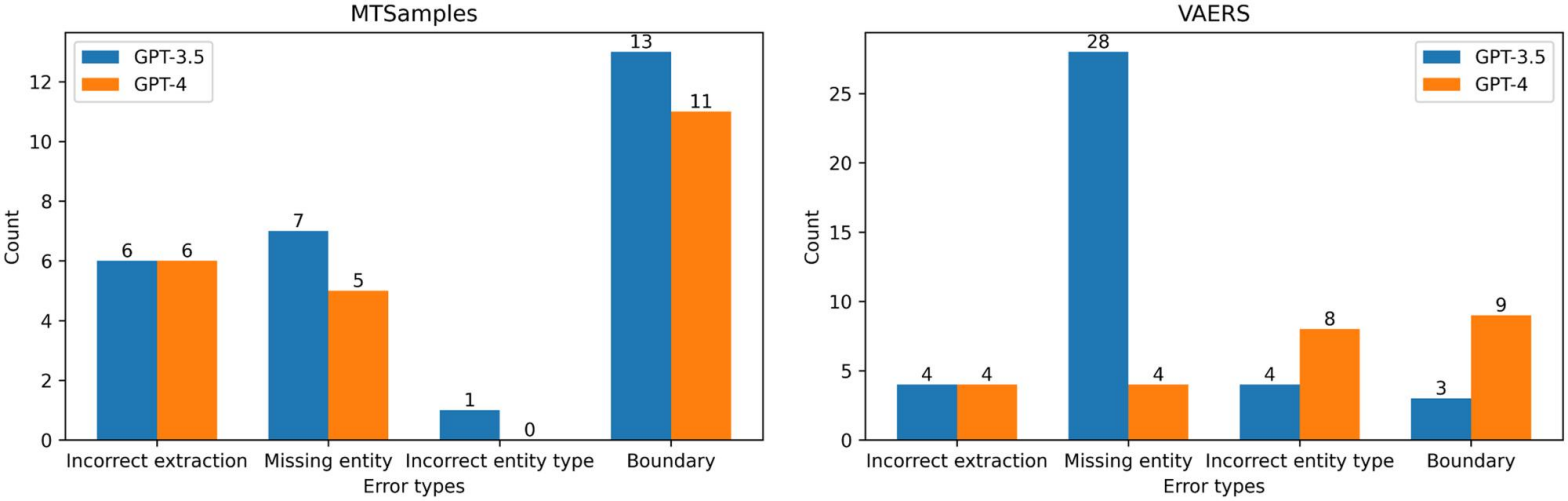
Summary of different error types with the number of occurrences and percentages.

A random sample of 20 sentences was selected from the outputs generated by each GPT model across the 2 datasets, postprocessing. This selection included sentences with both false positives and false negatives. The error analysis was conducted based on exact match. The error statistics derived from this analysis are presented in Figure 5. When assessed on a dataset basis, GPT-3.5 and GPT-4 exhibited similar error patterns for the MTSamples dataset. Both models encountered challenges when it came to identifying correct entity boundaries. This typically involved making decisions on whether to include article words (such as “the” in the phrase “the study drug”) or modifiers (such as “another large” in the phrase “another large stroke”) that precede a noun phrase. In assessing model performance, we considered the exact-match criteria, which may present a different challenge for GPT models compared to BioClinicalBERT. While BioClinicalBERT is fine-tuned specifically on annotated entities with clear boundaries, the GPT models, being LLMs, are trained on a broader and more diverse corpus. This distinction could impact their ability to adhere strictly to the exact boundaries of entities as defined in the training data, especially in the context of clinical NER where the linguistic structure and terminology are highly specialized.

As for the VAERS dataset, several factors may contribute to its increased complexity. Firstly, inner-annotator agreement was lower compared to the MTSamples dataset (ie, average F1 0.7707^31^ vs 0.8620), indicating less consistency in annotations. Additionally, the VAERS dataset contains more semantically specific annotation categories, such as distinguishing between different types of adverse events. This specificity demands a higher level of contextual understanding from the models. On the other hand, GPT-4's major difficulties lie in determining the correct entity boundaries and accurately classifying the entity types. This discrepancy can be attributed to the unique characteristics of each dataset. The VAERS dataset contains more complex entities (ie, Nervous adverse events vs Other adverse events) compared to the MTSamples dataset, leading to a higher error rate in entity type classification for the models. Another possible reason could be the inconsistency^31^ in annotation, which needs further investigation.

## Discussion

Our study hints at the as-yet unrealized potential of LLMs in clinical NER tasks by proposing a clinical task-specific prompt framework that incorporates annotation guidelines, error analysis-based instructions, and few-shot examples. We found that the performance of GPT models improved with the task-specific prompts. The best performance achieved by GPT-4 shows a competitive performance as that of BioClinicalBERT in the relaxed-match criteria.

LLMs are making paradigm-shifting changes in NLP research and development. Our finding shows a quick and easy path to build more generalizable clinical NER systems by leveraging LLMs. This will significantly change our current practice in clinical NLP. Traditionally, to build a machine learning or deep learning-based NER system for specific types of clinical entities, we have to build an annotated corpus of clinical documents, which is time-consuming and costly, as it often requires medical domain experts. Remarkably, our research shows that LLMs, devoid of further model training or fine-tuning, have exhibited exceptional performance. With merely 1- or 5-shot annotated samples, these models can achieve performance that is close to the fine-tuned models that require hundreds of training samples. This suggests a potential reduction in some of the costs associated with clinical NER system development, particularly in the areas of data annotation. However, it is important to note that this does not eliminate the need for expert input in creating annotation guidelines and in the initial phases of model training. While our study demonstrates that GPT models can achieve competitive performance with fewer annotated examples compared to traditional NLP systems, the role of subject matter experts remains crucial. Experts are needed to write precise annotation guidelines, perform initial annotations for error analysis and example generation, and validate the model's performance. Although the GPT models require fewer annotated instances, the costs associated with expert involvement, API usage, and running an LLM service should not be overlooked. A comprehensive comparison of resource requirements and costs between traditional NLP systems, word embedding models, and LLM-based systems would be valuable for future studies. This will provide a clearer understanding of the practical implications and feasibility of deploying LLMs in clinical NER tasks.

Moreover, our approach is generalizable—it shows consistent performance improvements across 2 different clinical NER tasks. The emergent abilities of LLMs^36^ have been further demonstrated in multiple clinical NER tasks here, indicating the feasibility of building 1 large model for diverse information extraction tasks in the medical domain, which is very appealing.

With those changes in mind, an urgent need will be to redesign the workflow for developing clinical NER systems using LLMs. The prompt framework for those 2 clinical NER tasks is the first step toward this direction and it sheds some lights for several aspects that are worth considering. The first aspect is how to clearly define an information extraction task. Our experiments show that incorporating annotation guidelines in prompt is very helpful in improving performance, which indicates medical knowledge (either in a knowledge base or from human experts) are still critical in LLM-based NER systems and how to obtain and represent task-specific knowledge in prompts need further investigation. We also demonstrated that supplying annotated examples is effective for performance improvement. Nevertheless, how to select informative and representative samples has not been investigated in this study and other advanced few-shot learning algorithms could be explored.

Another important issue is evaluation. In this study, we instructed GPT models to output entities following traditional NER approaches so that we can evaluate them using the previous evaluation scripts. However, we would argue that the current evaluation schema for NER may not be ideal for LLM-based systems. GPT models, due to their generative nature and extensive pretraining on diverse text corpora, exhibit a nuanced understanding of context and language structure. This enables them to interpret and generate text in a way that sometimes extends beyond the strict boundaries of predefined entity classes. For instance, GPT models often recognized lab tests with abnormal values (eg, “a blood sugar level of 40” or “white blood cell count of 23,500”) as medical problems. While this interpretation is contextually relevant and clinically meaningful, it deviates from the strict entity definitions used in our evaluation, leading to apparent mismatches. Therefore, a better evaluation schema would be needed to assess LLM performance more accurately.

Despite the promising results, our study has some limitations. First, we limited LLMs to GPT models in this study. In future, we will include other popular LLMs such as LLaMA and Falcon.^37–39^ Second, our few-shot learning approaches were relatively simple, and we plan to investigate other approaches such as the chain-of-thoughts method,^40–42^ hoping to yield better results.

## Conclusion

This is one of the first studies that systematically investigated GPT models for clinical NER via prompt engineering. In this study, we proposed a clinical task-specific prompt framework by incorporating annotation guidelines, error analysis-based instructions, and annotated samples via few-shot learning, and our evaluation on 2 clinical NER tasks shows that the GPT-4 model with our proposed prompts achieved close performance as the state-of-the-art BioClinicalBERT model. The best performance achieved by GPT-4 with 5-shot learning did not work as well as the BioClinicalBERT model on MTSamples and VAERS datasets. Nevertheless, considering that almost no training data was used in GPT models, their performance is already impressive, which hints the potential of LLMs in clinical NER tasks. While the results demonstrate a promising direction, they also underscore the need for further refinement and development before LLMs can consistently outperform established models like BioClinicalBERT in these specific applications.

## Supplementary Material

ocad259_Supplementary_Data

## Data Availability

Our code and datasets are available at: https://github.com/BIDS-Xu-Lab/Clinical_Entity_Recognition_Using_GPT_models
