# Supplementary material for: Improving large language models for clinical named entity recognition via prompt engineering
Source: J Am Med Inform Assoc. 2024 Jan 27;31(9):1812–20. doi: 10.1093/jamia/ocad259 (PMC11339492; doi:10.1093/jamia/ocad259)
Supplement: ocad259_Supplementary_Data [file ocad259_supplementary_data.docx]

# Supplementary Information:

**Improving Large Language Models for Clinical Named Entity Recognition via Prompt Engineering**

Yan Hu, MS^1^, Qingyu Chen, PhD^2,3^, Jingcheng Du, PhD^1^, Xueqing Peng, PhD^2^, Vipina Keloth, PhD^2^, Xu Zuo, MS^1^, Yujia Zhou, MS^1^, Xiaoqian Jiang, PhD^1^, Zhiyong Lu, PhD^3^, Kirk Roberts, PhD^1^, Hua Xu, PhD^2^

^1^ School of Biomedical Informatics, University of Texas Health Science at Houston, Houston, USA

^2^ Section of Biomedical Informatics and Data Science, School of Medicine, Yale University, New Haven, USA

^3^ National Center for Biotechnology Information, National Library of Medicine, National Institutes of Health, Maryland, USA

Corresponding Author: Hua Xu, PhD, FACMI, Section of Biomedical Informatics and Data Science, School of Medicine, Yale University, 100 College St, New Haven, CT 06510, USA; hua.xu@yale.edu

**1 Supplementary Materials**

**1.1 Complete prompts for two datasets**

**1.1.1 The 2010 i2b2 concept extraction task**

### Task

Your task is to generate an HTML version of an input text, marking up specific entities related to healthcare. The entities to be identified are: 'medical problems', 'treatments', and 'tests'. Use HTML <span> tags to highlight these entities. Each <span> should have a class attribute indicating the type of the entity.

### Entity Markup Guide

Use <span class="problem"> to denote a medical problem.

Use <span class="treatment"> to denote a treatment.

Use <span class="test"> to denote a test.

Leave the text as it is if no such entities are found.

### Entity Definitions

Medical Problems are defined as: phrases that contain observations made by patients or clinicians about the patient’s body or mind that are thought to be abnormal or caused by a disease. They are loosely based on the UMLS semantic types of pathologic functions, disease or syndrome, mental or behavioral dysfunction, cellormolecular dysfunction, congenital abnormality, acquired abnormality, injury or poisoning, anatomic abnormality, neoplastic process, virus/bacterium, sign or symptom, but are not limited by UMLS coverage.

Treatments are defined as: phrases that describe procedures, interventions, and substances given to a patient in an effort to resolve a medical problem. They are loosely based on the UMLS semantic types therapeutic or preventive procedure, medical device, steroid, pharmacologic substance, biomedical or dental material, antibiotic, clinical drug, and drug delivery device. Other concepts that are treatments but that may not be found in UMLS are also included. Treatments that a patient had, will have, may have in the future, or are explicitly mentioned that the patient will not have are all marked as treatments.

Tests are defined as: phrases that describe procedures, panels, and measures that are done to a patient or a body fluid or sample in order to discover, rule out, or find more information about a medical problem. They are loosely based on the UMLS semantic types laboratory procedure, diagnostic procedure, but also include instances not covered by UMLS.

### Annotation Guidelines

Only complete noun phrases (NPs) and adjective phrases (APs) should be marked. Terms that fit concept semantic rules, but that are only used as modifiers in a noun phrase should not be marked.

Include all modifiers with concepts when they appear in the same phrase except for assertion modifiers.

You can include up to one prepositional phrase (PP) following a markable concept if the PP does not contain a markable concept and either indicates an organ/body part or can be rearranged to eliminate the PP (we later call this the PP test).

Include articles and possessives.

Conjunctions and other syntax that denote lists should be included if they occur within the modifiers or are connected by a common set of modifiers. If the portions of the list are otherwise independent, they should not be included. Similarly, when concepts are mentioned in more than one way in the same noun phrase (such as the definition of an acronym or where a generic and a brand name of a drug are used together), the concepts should be marked together.

Concepts should be mentioned in relation to the patient or someone else in the note. Section headers that provide formatting, but that are not specific to a person are not marked.

### Error-analysis-based Guidelines:

Vital signs or vital signs with abnormal readings should be annotated as tests.

Medical specialists, services, or healthcare facilities should not be annotated, even if they might seem to fit into the categories of 'tests', 'treatments', or 'medical problems'. These entities are part of the healthcare delivery system and do not directly denote a test, treatment, or medical problem.

Consultation procedures should not be considered as tests.

### Examples

Example Input1: At the time of admission , he denied fever , diaphoresis , nausea , chest pain or other systemic symptoms .

Example Output1: At the time of admission , he denied <span class="problem">fever</span> , <span class="problem">diaphoresis</span> , <span class="problem">nausea</span> , <span class="problem">chest pain</span> or other systemic symptoms .

Example Input2: He had been diagnosed with osteoarthritis of the knees and had undergone arthroscopy years prior to admission .

Example Output2: He had been diagnosed with <span class="problem">osteoarthritis of the knees</span> and had undergone <span class="test">arthroscopy</span> years prior to admission .

Example Input3: After the patient was seen in the office on August 10 , she persisted with high fevers and was admitted on August 11 to Cottonwood Hospital .

Example Output3: After the patient was seen in the office on August 10 , she persisted with <span class="problem">high fevers</span> and was admitted on August 11 to Cottonwood Hospital .

Example Input4: HISTORY OF PRESENT ILLNESS : The patient is an 85 - year - old male who was brought in by EMS with a complaint of a decreased level of consciousness .

Example Output4: HISTORY OF PRESENT ILLNESS : The patient is an 85 - year - old male who was brought in by EMS with a complaint of <span class="problem">a decreased level of consciousness</span> .

Example Input5: Her lisinopril was increased to 40 mg daily .

Example Output5: <span class="treatment">Her lisinopril</span> was increased to 40 mg daily .

### Input Text: {}

### Output Text:

**1.1.2 The nervous system disorder-related event extraction task**

### Task

Your task is to generate an HTML version of an input text, marking up specific entities related to healthcare. The entities to be identified are: 'investigations', 'nervous adverse events', 'other adverse events', and 'procedures'. Use HTML <span> tags to highlight these entities. Each <span> should have a class attribute indicating the type of the entity.

### Entity Markup Guide

Use <span class="investigation"> to denote an investigation.

Use <span class="nervous_AE"> to denote a nervous adverse event.

Use <span class="other_AE"> to denote an other adverse event.

Use <span class="procedure"> to denote a procedure.

If no entity found, leave the text as it is.

### Entity Definitions

Investigation includes typical lab tests or examinations in the report, such as physical examination, oxygen saturation, electromyogram, etc.

Nervous adverse event includes typically nervous system-related problems, such as guillain-barré syndrome, ataxia, areflexia, hypoaesthesia, paraesthesia, dizziness, headache and other nervous system disorders.

Other adverse event includes medical problems that are assigned to other MedDRA SOCs, including gastrointestinal disorders, cardiac disorders, psychiatric disorders, musculoskeletal and connective tissue disorders, etc.

Procedure includes non-medical problem events such as individual immunization complications or related medical events (each immunization should be marked separately), surgeries such as catheter placement, hospitalization, emergence care, intubation, etc. A procedure refers to a specific medical or surgical activity carried out to diagnose, treat, or monitor a condition. Routine care activities or general healthcare administration such as 'sick call', 'doctor's visit', 'general checkup', etc. without a specific associated procedure or event should not be considered as a procedure. Note that 'vaccines administered' in absence of any complications or related medical events should not be considered a procedure.

Please note that in the case of negation where a certain adverse event, investigation, or procedure is clearly indicated NOT to have occurred (e.g., 'No bowel or bladder symptoms'), do not mark the entity.

### Annotation Guidelines

Only annotate events that already occurred (i.e., occurred before the diagnosis of GBS).

When annotating events related to Flu-GBS, do not include prepositions including modifiers of the event.

Separate events in discontinuous segments.

When annotating events, more generalized events should not be annotated.

When annotating events related to symptom improvement / progress or negation events, the following guideline should be used. In the case where the patient reported a specific adverse event first, and then reported improvement / progress of the adverse event, we should annotate it as an improved symptom. However, we do NOT need to annotate the negation of a symptom which the patient never reported before.

Events reported as history (events that did not happen to the reporting patient) should be annotated. Family history is important for risk prediction and may be included as a baseline information (e.g., for statistical analysis).

Some VAERS reports have duplicate events reported. For example, the same events / text are repeated twice in the report. The case we are interested in, is the recurrence of some adverse event, i.e., it requires the adverse event appears, then disappear, and then come back. In this case it should definitely be annotated twice. Additionally, we need to annotate the relief/improvement of the event if it is mentioned in the report. When no such information to decide whether it is a recurrence, the principle is that if there are multiple time stamps of the same event, we annotate it twice, if not, we can just keep one record.

### Error-analysis-based Guidelines:

When annotating events related to hospital admissions, transfers, or discharges, consider them as procedures. Specifically, annotate the words 'hospital', 'rehabilitation center', or any other healthcare facility involved in the patient's care as a procedure.

All abnormal symptoms should be considered as adverse events.

### Examples

Example Input1: Received flu shot 11 / 1 / 06 .

Example Output1: Received <span class="procedure">flu shot</span> 11 / 1 / 06 .

Example Input2: 1 / 28 / 05 PM : ascending redness left elbow then from fingertips .

Example Output2: 1 / 28 / 05 PM : <span class="other_AE">ascending redness left elbow then from fingertips</span> .

Example Input3: Unable to stand due to severe ataxia .

Example Output3: <span class="nervous_AE">Unable to stand</span> due to severe <span class="nervous_AE">ataxia</span> .

Example Input4: At 4 AM on 12 - 16 - 11 got up again to go to the bathroom and on the way out my right leg gave out from under me again and my husband saw me and tried to help me and then both legs wouldn ' t work .

Example Output4: At 4 AM on 12 - 16 - 11 got up again to go to the bathroom and on the way out my <span class="nervous_AE">right leg gave out</span> from under me again and my husband saw me and tried to help me and then <span class="nervous_AE">both legs wouldn ' t work</span> .

Example Input5: Seen by neurologist and diagnosed with Guillain Barre Syndrome .

Example Output5: Seen by neurologist and diagnosed with <span class="nervous_AE">Guillain Barre Syndrome</span> .

### Input Text: {}

### Output Text:

- 1. **Learning Curve of BioClinicalBERT on the validation sets**

To provide additional insights into model training and validation, we conducted a learning curve analysis for both the MTSamples and VAERS datasets using BioClinicalBERT. The learning curves, depicted in Figures S1, illustrate the model's performance over epochs on the validation set. For MTSamples, the F1 score improved sharply in the initial epochs, and plateaued around epoch 5. In the case of VAERS, the improvement in F1 was also sharp in the beginning and leveled off near epoch 4, maintaining a consistent score thereafter. These trends suggest that the model reached its performance capacity quickly and did not exhibit signs of overfitting, as evidenced by the stable F1 scores beyond the plateau point.


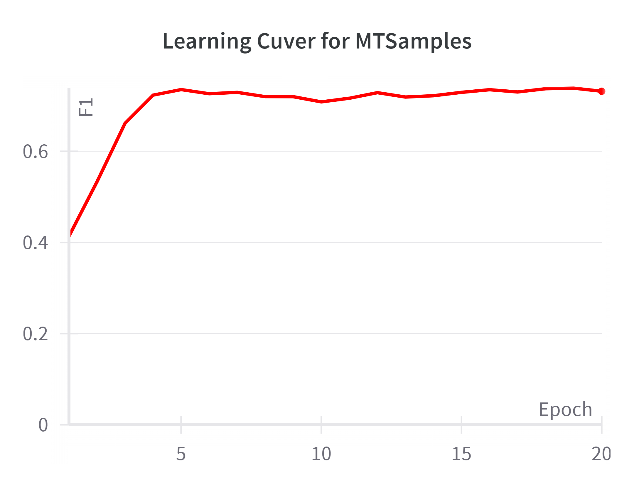

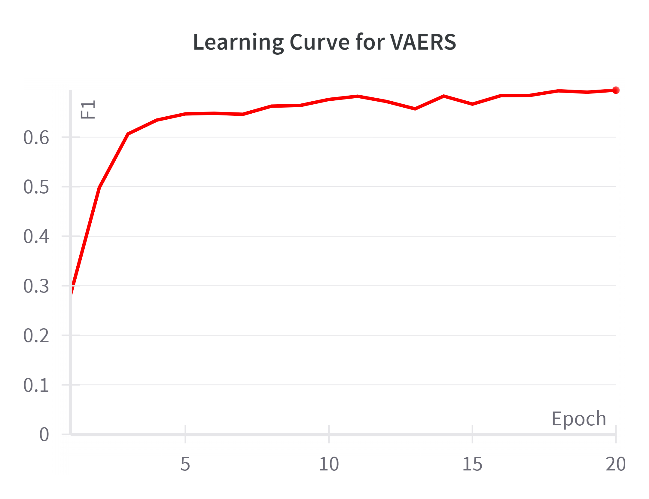


**Figure S1.** Learning curve of BioClinicalBERT on validation sets across epochs for MTSamples and VAERS datasets
